# Regulation of Airway Epithelial-Derived Alarmins in Asthma: Perspectives for Therapeutic Targets

**DOI:** 10.3390/biomedicines12102312

**Published:** 2024-10-11

**Authors:** Ravneet K. Hansi, Maral Ranjbar, Christiane E. Whetstone, Gail M. Gauvreau

**Affiliations:** Division of Respirology, Department of Medicine, McMaster University, Hamilton, ON L8N 3Z5, Canada; hansir@mcmaster.ca (R.K.H.); ranjbm1@mcmaster.ca (M.R.); whetstoc@mcmaster.ca (C.E.W.)

**Keywords:** asthma, airway epithelium, alarmin cytokines, allergies, respiratory infections, pollutants, biologic therapy

## Abstract

Asthma is a chronic respiratory condition predominantly driven by a type 2 immune response. Epithelial-derived alarmins such as thymic stromal lymphopoietin (TSLP), interleukin (IL)-33, and IL-25 orchestrate the activation of downstream Th2 cells and group 2 innate lymphoid cells (ILC2s), along with other immune effector cells. While these alarmins are produced in response to inhaled triggers, such as allergens, respiratory pathogens or particulate matter, disproportionate alarmin production by airway epithelial cells can lead to asthma exacerbations. With alarmins produced upstream of the type 2 inflammatory cascade, understanding the pathways by which these alarmins are regulated and expressed is critical to further explore new therapeutics for the treatment of asthmatic patients. This review emphasizes the critical role of airway epithelium and epithelial-derived alarmins in asthma pathogenesis and highlights the potential of targeting alarmins as a promising therapeutic to improve outcomes for asthma patients.

## 1. Introduction

Asthma is a chronic respiratory condition that affects up to 300 million people worldwide, making it one of the most common respiratory diseases [1]. This condition is characterized mainly by airway hyperresponsiveness and inflammation, largely attributed to airway dysfunction and an altered immune response. Often, this results in an exaggerated type 2 (T2) inflammatory phenotype [1,2]. Central to this phenotype is the release of T2 cytokines, such as interleukin (IL)-4, IL-5, and IL-13, by T helper 2 (Th2) cells and group 2 innate lymphoid cells (ILC2s), resulting in elevated levels of immunoglobulin (Ig)E and eosinophilia [3,4,5]. These immunological markers not only perpetuate an allergic inflammatory response but also induce pathological changes in the airways [4,5].

For many asthmatics, inhaled corticosteroids (ICSs) are the standard means of treatment to effectively reduce the production of inflammatory mediators by activating glucocorticoid receptors. Through a reduction in T2 cytokines and cellular inflammation including sputum eosinophil numbers, ICS treatment also helps to manage airway hyperresponsiveness in mild asthmatics [6]. However, despite their widespread use, over 20% of asthmatics find insufficient relief from ICS treatment due to their severe or difficult-to-treat manifestations of the disease, putting them at risk for exacerbations [1]. This treatment disparity underscores the necessity to further explore the underlying mechanisms that propagate airway inflammation, which may not be adequately targeted by corticosteroids.

The airway epithelium acts as a sentinel interface to protect against inhaled contaminants. Its fundamental role in maintaining airway homeostasis has been increasingly recognized, with growing evidence implicating epithelial dysfunction in the pathophysiology of various respiratory diseases, including asthma [1]. Notably, epithelial-derived alarmin cytokines are recognized to be master regulators of the T2-skewed inflammatory phenotype in asthma [7]. Thymic stromal lymphopoietin (TSLP), IL-33, and IL-25, in particular, are critical upstream mediators in the initiation of this inflammatory cascade [8]. With their integral role in the pathogenesis of asthma, understanding how alarmins are regulated in the airways is critical in elucidating potential pathways for therapeutic targets. This review will explore the mechanisms regulating alarmin cytokine expression in the airway epithelium, the contribution of alarmin cytokines to T2 inflammation and asthma pathogenesis, and their role as targets of therapeutic strategies for asthma management.

## 2. The Airway Epithelium: Structure and Function

The lungs and airways are perpetually exposed to the external environment, rendering them vulnerable to the effects of a wide range of aeroallergens, chemicals, and particulate matter present in inspired air. The airway epithelium is a layer of specialized cells lining the respiratory tract, serving as the primary point of contact for inhaled particulates and pathogens and functioning as both a structural and immunological barrier [9]. The epithelium forms a tightly regulated barrier to physically inhibit the paracellular transport of foreign pathogens to the underlying mucosa. This barrier structure is facilitated by a network of specialized intercellular adhesion molecules, including tight and adherens junctions, ensuring that epithelial cells adhere to one another and the basement membrane [10]. The luminal surface of the upper airway epithelium is primarily comprised of ciliated cells and goblet cells. Together, these cells form the epithelial mucociliary clearance mechanism, which utilizes mucus secretion by goblet cells to trap particles while the beating of ciliated cells expels the mucus-entrapped particulates out of the respiratory tract [10]. Beyond these structural defenses, the airway epithelium plays a crucial role as an immunological barrier, actively detecting and responding to inhaled pathogens and pollutants.

In line with its critical role in orchestrating the initial immune responses, the airway epithelium expresses a wide range of pattern recognition receptors (PRRs). These receptors recognize pathogen-associated molecular patterns (PAMPs) from inhaled pathogens, including allergens and parasites, and host-derived damage-associated molecular patterns (DAMPs) released by dying or damaged cells. Upon activation, these PRRs initiate host defense mechanisms through the rapid release of antimicrobial peptides and enzymes, such as lysozyme and defensins, and various cytokines, including alarmin cytokines, to rapidly initiate cellular communication [11]. Key PRRs involved in epithelial immune response include Toll-like receptors (TLRs), protease-activated receptors (PARs), C-type lectin receptors (CLRs), retinoic acid-inducible gene (RIG)-I-like receptors (RLRs), nucleotide-binding oligomerization domain (NOD)-like receptors (NLRs) and purinergic receptors. While pathogens can be detected by multiple different PRRs, each class plays a specific role in recognizing distinct types of molecules on pathogens and allergens.

TLRs are the most extensively studied class of PRRs in the pathology of asthma. There are a total of 10 different human TLRs that are categorized as either being expressed on the cell surface (TLRs 1, 2, 4–6, and 10) or intracellularly within endosomal compartments (TLRs 3 and 7–9) [12]. Cell surface TLRs primarily detect bacterial and some fungal lipid components, whereas intracellular TLRs are responsible for the detection of viral nucleic acids [11]. PARs are important G-protein-coupled receptors that require proteolytic cleavage for activation, making them key in the detection of various proteases, such as tryptase produced by mast cells [13]. Complex carbohydrates, like glycans, are recognized by CLRs due to their carbohydrate-binding domains. While the expression of CLRs on airway epithelial cells is less characterized, they do express dectin-1 and the mannose receptor (MR), which are important CLRs responsible for detecting β-glucans found in fungal cell walls and high mannose glycans on the surface of pathogens, respectively [14]. RLRs, including RIG-I and melanoma differentiation factor 5 (MDA-5), are a family of cytoplasmic PRRs that are known to be the principal sensors of viral RNA [15]. NLRs recognize microbial components, including bacterial peptidoglycan, and stress signals [16]. Like RLRs, these receptors are also expressed intracellularly. While NOD1 and NOD2 work as receptors, NLRP3 differs in that it serves as a cytoplasmic sensor protein that forms part of the multi-protein inflammasome complexes involved in activating IL-1β [17]. In contrast to the rest of the PRRs, purinergic receptors like P2X7 and P2Y2 are primarily activated by adenosine and extracellular nucleotides, like ATP released by damaged cells or cells that are under stress [18]. Given that the activation of various PRRs by triggers represents the most upstream, even facilitating host immune response, understanding how alarmin expression is regulated by these pathways is crucial for identifying potential targets for asthma treatment.

## 3. The Cell-Derived Alarmins IL-25, IL-33 and TSLP and Their Role in Asthma Pathology

### 3.1. IL-25, IL-33 and TSLP Alarmin Expression and Signaling

Asthmatic airways exhibit markedly upregulated alarmin expression levels, suggesting that the airway epithelium is more responsive to environmental stimuli. Among these, TSLP, IL-25, and IL-33 play pivotal roles as upstream regulators, initiating and amplifying inflammatory pathways that drive asthma symptoms, making them critical targets for therapeutic interventions. TSLP, a member of the IL-2 family, is released by a variety of cells including epithelial cells, dendritic cells (DCs) and basophils [11]. It signals through the heterodimer TSLP receptor consisting of TSLPR and IL-7Rα [8]. TSLP is expressed as one of two main isoforms: short TSLP, which is primarily found to be expressed during basal conditions, and long TSLP, whose expression is induced by inflammatory stimuli [11]. TSLP is largely expressed at barrier surfaces in response to various danger signals [11]. Endobronchial tissues from asthmatic patients at baseline exhibit an increased number of epithelial cells expressing TSLP mRNA compared to non-asthmatic controls [19]. Increased TSLP concentration in asthmatic patients correlates with disease severity and the risk of further asthma exacerbations [19,20]. Moreover, animal studies have demonstrated an overexpression of TSLP in mice lungs with both severe airway inflammation and hyperresponsiveness [21]. IL-25 is one of six members of the IL-17 family, which also includes IL-17A, IL17B, IL-17C, IL-17C, and IL-17F [11]. IL-25R, composed of IL-17RA and IL-17RB subunits as a disulphide-linked dimer, is the primary receptor that this alarmin signals through [11]. While IL-25 is widely expressed by a wide range of cell types, eosinophils and basophils have been demonstrated to be a major source of IL-25 in asthmatic patients [11]. IL-33, on the other hand, is a member of the IL-1 family and is signaled through the heteromeric receptor consisting of the primary receptor, the suppression of tumerigenicity 2 (ST2) and the co-receptor IL-1 receptor accessory protein (IL-1RAcP) [22]. This alarmin is constitutively expressed in the nuclei of various cells, including epithelial cells, fibroblasts, and airway smooth muscle cells [11]. Interestingly, the expression of both IL-33 and IL-25 is elevated in the bronchial biopsies of asthmatic patients, correlating with the disease severity [23,24]. Allergen challenges significantly amplify the expression of baseline levels of all three alarmin cytokines in the bronchial epithelium of individuals with mild atopic asthma [25], highlighting how environmental triggers can exacerbate alarmin cytokine levels in asthmatic airways. Increased alarmin expression is also observed in the upper airways of asthmatics with comorbid allergic rhinitis (AR), correlating with disease severity [26,27,28]. However, it is important to note that while the respiratory tract is continuous, alarmin expression in the lower respiratory tract does not necessarily coincide with that in the upper respiratory tract. Specifically, the lower respiratory tract of allergic individuals expresses higher levels of IL-33 and TSLP compared to the upper respiratory tract [29,30], suggesting a variance in the sensitivity of the epithelium to triggers in different areas of the airway in asthmatic individuals.

### 3.2. Alarmin Cytokines Drive Type 2 Inflammation

This heightened expression of alarmin cytokines plays a critical role in the pathophysiology of asthma. Alarmins IL-25, IL-33 and TSLP are released by airway epithelial cells, following exposure to environmental triggers, and stimulate innate immune cells including ILC2s, eosinophils, basophils, mast cells and DCs. Alarmins, especially TSLP, which is referred to as the master switch of immunity, are crucial for the development and regulation of ILC2 and Th2 proliferation, widely known as the ILC2-DC-Th2 axis [31]. IL-25, IL-33 and TSLP individually, and synergistically, directly bind to and induce ILC2 activation and the production of T2 cytokines (IL-4, IL-5, and IL-13). While IL-33 plays a critical role in ILC2 activation, in combination with IL-2 or TSLP, it can further amplify cytokine production [32]. IL-33 causes the activation of ILC2s by upregulating co-stimulatory molecules OX40L and PD-L1, thereby inducing ILC2s to produce IL-5 and IL-13 [33,34]. On a per-cell basis, IL-33 is able to induce approximately 10-fold more IL-5 and IL-13 from ILC2s compared to activated Th2 lymphocytes [35]. Alone, IL-25 stimulates the ILC2 population to rapidly secrete chemokines including thymus and activation-regulated chemokine (TARC), eotaxin and macrophage-derived chemokine (MDC), which can contribute to airway remodeling and angiogenesis by increasing endothelial cell vascular endothelial growth factor (VEGF)/VEGF receptor expression [36]. However, in conjunction with the co-stimulatory cytokine IL-2, IL-25 induces the well-characterized proliferation of T2 cytokines, IL-4, IL-5, and IL-13 [37]. IL-4 production by ILC2s drives the differentiation of naïve T helper type 0 (T_H_0) lymphocytes into Th2 lymphocytes, causing the further release of IL-4, IL-5, IL-9, and IL-13, but not interferon-gamma (IFN-γ), from Th2 cells, further skewing the inflammatory reaction towards a T2 response [38,39]. ILC2-derived IL-5 helps to activate and recruit eosinophils, which contribute to inflammation and tissue damage by releasing cytotoxic mediators [38]. ILC2-derived IL-9 amplifies ILC2 survival and activation by stimulating mast cells to release IL-2 [40]. ILC2-derived IL-13 facilitates the migration of activated DC into draining lymph nodes, where naïve T cells are differentiated into Th2 cells, activating the switch to an adaptive immune response [41]. Additionally, IL-9 production by ILC2s promotes ILC2 expansion and regulates the release of amphiregulin, which plays an important role in tissue repair during lung inflammation [42,43]. Finally, Th2 cell-derived IL-2 induces ILC2 proliferation and IL-13 secretion, creating ILC2-Th2 cell crosstalk, which is complex and bidirectional, forming an inter-connected loop critical in rapidly producing an inflammatory response. In addition to ILC2s, alarmins IL-33 and TSLP can induce the release of cytokines IL-4 and INF-y from nature killer T cells [44,45]. Recent studies have also demonstrated that alarmins directly contribute to mucus hypersecretion in asthma. Research assessing the role of IL-33 in airway hyperreactivity has demonstrated that IL-33 is associated with goblet cell hyperplasia, leading to mucus overproduction [46]. Interestingly, blocking IL-33 activity has demonstrated a restorative effect on epithelial physiology, re-establishing the presence of ciliated cells over mucus-producing cells [47]. Furthermore, TSLP/IL-33 knockout mice treated with anti-IL-25 antibody during the initiation of allergic airway inflammation exhibit a reduction in mucus production, lung remodeling and inflammation [48]. These findings provide important clinical insights into the connection between alarmins and mucus hypersecretion in asthma pathogenesis.

Alarmin cytokines can also directly target effector cells involved in type 2 immunity, including eosinophils, basophils, and mast cells. IL-25 directly enhances eosinophil survival, recruitment, and migration to the site of inflammation [49,50,51,52] and facilitates endothelial transmigration by upregulating the expression of intercellular adhesion molecule (ICAM)-1 and suppressing ICAM-3 and L-selection, moreover exerting pro-inflammatory effects by driving the expression of chemokines monocyte chemoattractant protein-1 (MCP-1), macrophage inflammatory protein-1α (MIP-1α) and cytokines IL-8 and IL-6 [49,52]. IL-4-producing eosinophils, when cocultured with IL-33, can significantly increase ILC2 proliferation and augment the secretion of IL-5 and IL-13, thereby magnifying type 2 responses [53]. IL-33 induces eosinophil CD11b expression and cell adhesion by the upregulation of ICAM-1 and VCAM-1, as well as enhancing eosinophil survival and cytokine production in an IL-4-dependent manner [34,54,55,56]. TSLP stimulation elicits a dose-dependent release of critical eosinophil growth factors IL-5, IL-13, and GM-CSF from CD34+ core blood progenitor cells, while concurrently enhancing eosinophil survival by preventing apoptosis, and facilitates eosinophil extravasation and migration to sites of inflammation through the upregulation of CD18 and ICAM-1 [57,58,59].

Basophils and mast cells are responsive to alarmin cytokine stimulation. IL-25, IL-33 and TSLP act synergistically to augment basophil survival and migration while serving as potent mediators that amplify IgE-dependent and IgE-independent basophil degranulation, the release of histamine and the expression of cytokines [60,61]. IL-25 and IL-33 are known to induce the release of LTC_4_ and increase the expression of IL-3 and IL-13 in basophils [60,62]. IL-33 also induces the release of IL-4, IL-5, IL-6, IL-8, IL-9, IL-13, MCP and MIP from basophils [44,61,63,64], and TSLP induces the expression of IL-4 and IL-13 [65]. TSLP-stimulated mast cells release a broad spectrum of cytokines including IL-5, IL-13, IL-6, IL-8, IL-10, and GM-CSF and chemokines CXCL8 and CCL1 while suppressing TGF-beta [66]. Additionally, mast cells have also been shown to regulate epithelial TSLP expression, suggesting the existence of a regulatory feedback loop between mast cells and epithelial cells [67]. Although mast cells are known to express the IL-25 receptor, its precise function remains unclear [68]. Mast cells and basophils play critical roles in modulating ILC2s. Basophil-derived IL-4 enhances ILC2 sensitivity to alarmin cytokines and promotes ILC2 migration to sites of inflammation [69]. Interestingly, mast cells are not only activated by alarmin cytokines but contribute to type 2 immune responses by producing IL-25, IL-33 and TSLP themselves, further activating ILC2 and Th2 cells. Mast cell-derived IL-33 fosters ILC2 expansion and the production of IL-13 and IL-9, which in turn are pivotal in promoting mast cell proliferation and stimulation [70,71]. IL-33-activated DCs prolong the survival of mast cells, enhancing adhesion and stimulating cytokine production from mast cells [72].

As a pivotal cell bridging the innate and adaptive immune system, the DC responds directly to TSLP by upregulating the co-stimulatory molecules CD40, CD80, CD86, and OX40L. This upregulation drives the differentiation of Th2 cells and Th9 cells, promoting their production of type 2 cytokines [73,74,75]. TSLP induces the release of TARC/CCL17, DC-CK1/pulmonary and activation-regulated chemokine (PARC)/CCL18, MDC/CCL22, and MIP3b/CL19 [76]. Meanwhile, IL-33 activates resident dendritic DC, enabling them to promote the differentiation of naïve CD4+ T cells into polarized Th2 cells. IL-33 also acts directly on CD4+ T cells, inducing the secretion of cytokines IL-4, IL-5, IL-13, and IL-9 [63,77]. Conversely, IL-25 enhances the secretion of T2 cytokine from TSLP-DC-activated Th2 memory cells by upregulating the production of T2 transcription factors in an IL-4-independent manner [78]. Notably, Th2 cells also negatively regulate IL-33-induced cytokine production, adding complexity to this regulatory network [79]. Although macrophages are not traditionally emphasized in T2 inflammation, emerging evidence suggests alarmins activate cytokine release in macrophages, contributing to downstream effects of T2 inflammation. IL-25 mediates a negative feedback loop between epithelial cells and macrophages, attenuating the release of exosomes and reducing exosome-induced tumor necrosis factor α (TNF-α) expression from adjacent macrophages [80]. Additionally, TSLP promotes the differentiation and activation of alternatively activated macrophages (M2 macrophages) during allergic inflammation [81]. IL-33 further induces naïve macrophages to produce M1 chemokines such as CCL3 while also driving previously polarized macrophages to express M2-associated chemokine markers, including CCL17, CCL18, and CCL24, and stimulating alveolar macrophages to secrete IL-13 [82,83].

TSLP is a powerful regulator of the adaptive immune system. TSLP signaling directly influences naïve T cells by promoting their proliferation and differentiation into Th2 cells in the presence of TCR stimulation, largely through the induction of IL-4 gene transcription [84,85,86]. Additionally, TSLP can drive the proliferation and differentiation of regulatory T cells (Tregs) via its effects on DCs [87,88]. CD4+ T cells and CD8+ T cells typically do not respond to TSLP under resting conditions; however, their expression of TSLP receptors increases following their activation [89]. Beyond its effects on T cells, TSLP supports B-cell lymphopoiesis by promoting the proliferation and differentiation of multilineage-committed CD34+ progenitor cells, pro-B cells and pre-B cells [90,91]. Together, IL-25, IL-33, and TSLP orchestrate a network of complex interactions that not only drive the recruitment and activation of immune cells like ILC2s, eosinophils, basophils, and mast cells but also influence tissue remodeling and airway hyperresponsiveness. This multifaceted involvement highlights their central role in asthma pathology and underscores their potential as crucial therapeutic targets.

The alarmin cytokines IL-25, IL-33 and TSLP lead to the production of type 2 cytokines including IL-4, IL-5, IL-9 and IL-13, as described above. These type 2 cytokines support inflammatory pathways that are critically important in the pathophysiology of asthma. IL-4 promotes cellular inflammation through the interaction of VCAM-1 on the vascular endothelium, facilitating the direct migration of T lymphocytes, monocytes, basophils, and eosinophils, as well as increasing cell survival [92,93]. IL-4 induces the isotype switch and secretion of IgE by B cells and enhances IgE-mediated immune responses by upregulating low-affinity IgE receptors (FcεRII) on B cells and mononuclear phagocytic cells along with high-affinity IgE receptors (FcεRI) on mast cells and basophils [94]. IL-4 also inhibits eosinophil apoptosis and promotes the eosinophil release of chemotaxis and eotaxin [95]. It also induces mucin gene expression and the hypersecretion of mucus and the expression of eotaxin from fibroblasts [96,97]. In T2 immunity, IL-4 and IL-13 share a complex receptor composed of IL-4Rα and IL-13Ra1 subunits, and upon sharing 25% homology, IL-4 and IL-13 have overlapping but distinct functions. Similar to IL-4, IL-13 can induce B cell proliferation and class switching from IgG_4_ to IgE in the presence of CD40/CD40L [98]. Moreover, similar to the function of IL-4, IL-13 can induce the expression of FcεRII on B cells and the expression of FcεRI on mast cells [99,100]. IL-13 promotes eosinophil survival and the activation, and recruitment of eosinophils, stimulating peripheral blood eosinophils trafficking to the lung to produce IL-5 and eosinophil chemokines, like eotaxin [101,102]. IL-13 also has direct effects on structural cells such as inducing vascular cell adhesion molecule (VCAM)-1 on endothelial cells and the release of eotaxin from epithelial cells, both of which are important in eosinophil recruitment [103,104]. IL-13 increases muscle contraction through the increased expression of B1 integrin and VCAM-1 on lung fibroblasts in response to acetylcholine, a cholinergic-induced contraction of airway smooth muscle cells and collagen synthesis, contributing to airway remodeling [97,105,106]. Furthermore, IL-13 is a potent inducer of goblet cell metaplasia, invoking mucus hypersecretion [107,108]. It promotes the expression of mucins such as MUC5AC and MUC5B, contributing to mucus hypersecretion and airway obstruction, a key factor in asthma exacerbations [109,110]. IL-5 is the key cytokine for eosinophils, being the most important factor responsible for eosinophil differentiation, growth, recruitment to the airway, survival, and activation [111,112,113,114]. The IL-5 receptor (IL-5Rα) has been found on other granulocytes, basophils, neutrophils, and even mast cells; however, the effects of IL-5 on these cells are relatively understudied [115,116,117].

## 4. Epithelial-Derived Cytokines HMGB1, IL-1α, IL-36 and TL1A in Asthma Pathology

### 4.1. HMGB1

While TSLP, IL-25, and IL-33 are acknowledged as the key promoters of the T2-skewed inflammatory response in asthmatic airways, there is increasing evidence for the role of additional epithelial-derived cytokines in asthma pathology. Among these cytokines is high mobility group box 1 (HMGB1). HMGB1 is known to be localized in the nucleus of many cells, including airway epithelial cells, and is transported into the cytosol, where it accumulates in lysosomal-associated vesicles to be released via exocytosis upon stimulation with extracellular adenosine triphosphate (ATP) or lysophosphatidic acid (LPA) [118]. Clinical work demonstrates significantly higher levels of HMGB1 in the induced sputum and plasma of asthmatic patients compared to healthy controls [119,120]. Moreover, these increased levels inversely correlate with the percent predicted forced expiratory volume in 1 s (%FEV1) and positively correlate with the severity of asthma [120]. Higher HMGB1 levels in asthmatic sputum are associated with increased sputum eosinophils and IL-5 and IL-13 [121]. Interestingly, murine asthma models have also demonstrated that blocking HMGB1 activity attenuates pathological airway changes, including airway inflammation and hyperresponsiveness [121,122]. Moreover, both in vivo and in vitro work shows that HMGB1 can induce Th2 polarization by directly acting on CD4^+^ naïve T cells to induce Th2 cells [123], highlighting that HMGB1 may also play a critical role in propagating asthmatic T2 inflammation.

### 4.2. IL-1α

IL-1 alpha (IL-1α), an important pro-inflammatory cytokine that belongs to the IL-1 family, is constitutively expressed in immune and structural cells, including airway epithelial cells, and is critical in mediating innate and adaptive immune responses [124]. In the context of asthma, bronchoalveolar lavage fluid (BALF) and sputum from asthmatic individuals sensitized to fungi demonstrate elevated IL-1α levels [125]. Godwin et al. investigate this response further with an experimental mice model and demonstrate that the signaling of this cytokine via the IL-1 receptor (IL-1R) promotes neutrophilic inflammation and increased airway hyperreactivity, an effect that is attenuated when treated with an IL-1R antagonist [125]. Additionally, IL-1α is more pronounced in asthmatic primary airway epithelial cells, an effect that is linked to fibroblast-driven inflammation and the dysregulated repair of the asthmatic epithelial–mesenchymal tropic unit [126]. Pre-clinical studies in mice demonstrate that the IL-1α propagation of allergic inflammation is mediated by the activation of airway epithelial cells as mice lacking the IL-1R on non-hematopoietic cells fail to mount house dust mite (HDM)-induced Th2 responses and thus do not develop asthma [127], providing evidence that this cytokine is critical in the propagation of the allergic inflammation in asthma.

### 4.3. IL-36

IL-36, considered a novel member of the IL-1 family, is increasingly recognized for its role in immune homeostasis and inflammation. To date, there are five isoforms in the IL-36 family including IL-36α, IL-36β, IL-36γ, IL-36Ra, and IL-38, of which the first three work as inflammatory agonists that bind to the IL-36 receptor (IL-36R) and the latter two act as antagonists to inhibit inflammatory responses [128]. With this family of cytokines being relatively new, there is limited information on the role of IL-36 in asthma pathology. However, Li et al. demonstrate that asthmatic sputum had increased concentrations of IL-36α and IL-36β and decreased concentrations of IL-36Ra compared to non-asthmatic controls [129]. Interestingly, these levels varied in different asthma phenotypes, with significantly higher concentrations of IL-36α/β in neutrophilic asthmatics compared to the eosinophilic asthma group [129]. Another study demonstrated that serum from individuals with AR had an elevated expression of IL-36 cytokines and IL-36R, with these levels being especially prominent in those with asthma comorbidity [130]. Moreover, in vitro work demonstrates that stimulating human nasal epithelial cells with IL-25 and IL-33 promotes the mRNA expression of IL-36γ [130], suggesting that other alarmin cytokines can promote further cytokine propagation in allergic response. Interestingly, RNAseq data retrieved from the induced sputum of atopic asthmatic subjects before and after an inhaled allergen challenge demonstrated an increase in IL-36-related genes 24 h post allergen challenge, further providing evidence that this pathway may play a key role in the regulation of allergic airway inflammation [131]. However, more work is required to understand how IL-36 further propagates allergic inflammation and mediates asthma pathology.

### 4.4. TL1A

Recently, tumor necrosis factor (TNF)-like cytokine 1A (TL1A), belonging to the TNF family, has also been highlighted as an epithelial cell alarmin [132]. Schmitt et al. demonstrate that TL1A is constitutively expressed in both human and mouse epithelium and is rapidly released after allergen exposure [133]. TL1A is proposed to cooperate with IL-33 to induce IL-9^high^ ILC2s, termed ILC9, that go on to produce large amounts of IL-9, along with IL-5 and IL-13 [133]. Interestingly, compared to IL-33-activated ILC2s, this novel ILC9 phenotypic subset exhibits a greater ability to remain active in vivo and induce the IL-5-dependent allergic airway inflammatory response [133]. Furthermore, clinical work by Machida et al. demonstrates that the inhaled allergen challenge induces a significant increase in airway TL1A and sputum death receptor 3 (DR3)^+^ ILC2s in mild asthmatics [134]. Further in vitro work demonstrates that ILC2 stimulation with TL1A induces an increased expression of IL-5, which is reduced with the use of the corticosteroid dexamethasone [134]. Interestingly, the presence of both TL1A and TSLP induces persistent ILC2 IL-5 expression despite treatment with dexamethasone [134], suggesting that TSLP and TL1A can synergistically induce a persistent inflammatory state, even in the presence of corticosteroid treatment. Bronchial epithelial tissue biopsies from asthmatic individuals demonstrate a significantly increased expression of TL1A and its respective receptor, DR3 [135]. This increase in TL1A and DR3 is also mimicked in asthmatic mice lungs, where it is further demonstrated that this TL1A/DR3 axis not only contributes to airway inflammation and tissue destruction [136] but also mediates airway remodeling through the epithelial–mesenchymal transition (EMT), a biological process through which epithelial cells lose important characteristics, including barrier integrity and polarity, and transition into a mesenchymal state [135]. In vivo and in vitro work suggests that blocking this TL1A/DR3 axis through inhibiting TL1A improves this observed EMT in airway epithelial cells [135]. While further research is required to completely understand the underlying mechanisms of TL1A and its role in asthma pathology, this current research provides compelling evidence of TL1A as a novel alarmin, highlighting it as a potential therapeutic target.

## 5. Triggers That Mediate the Release of Alarmin Cytokines from Airway Epithelial Cells

### 5.1. Aeroallergens

**Figure 1:**
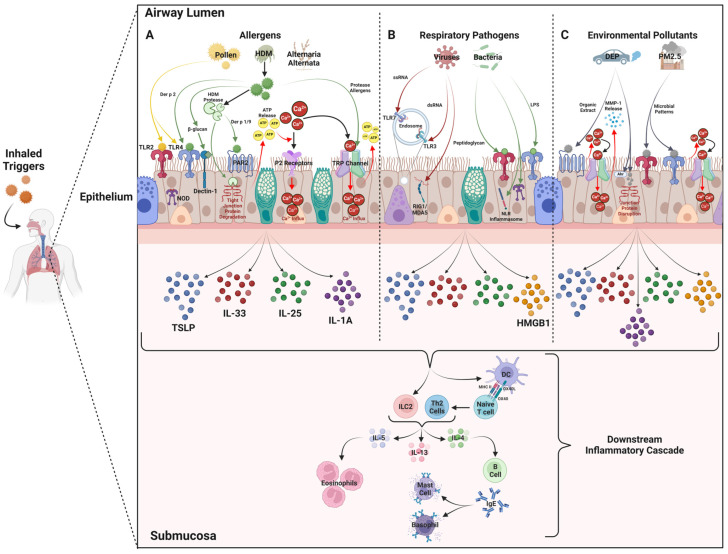
The airway epithelial production of alarmins in response to inhaled triggers. (**A**) Allergen triggers like pollen and HDM activate PRRs like TLR2 and 4 [137,138,139]. Protease allergens, including Der p 1 and 9 found in HDM, have the ability to directly disrupt and degrade tight junction proteins and can also trigger PAR2 [137,143,144]. Other motifs, like β-glucans and microbial patterns, can be detected by PRRs like dectin-1 and NOD1, respectively [137,140,142,145,146]. Allergen exposure can also induce the release of ATP into the extracellular space which, in turn, induces the influx of Ca^2+^ via P2 purinergic receptors [147]. TRP channels like TRPA1 and TRPV1 are also implicated in allergen-mediated Ca^2+^ influx, along with the release of ATP [148,149]. Allergen triggers via these receptors and channels induce the production of TSLP, IL-33, IL-25 and IL-1α [140,141,142,147,148,149,150,151,152,153]. (**B**) Viral pathogens are primary detected by intracellular TLRs located in endosomes, including TLR3 and TLR7, which can recognize viral double-strand (ds) RNA and single-strand (ss) RNA, respectively. The further detection of viral nucleic acids is mediated by cytosolic RLRs like RIG-1 and MDA-5 [154]. Bacterial pathogens are typically recognized by TLR2 and 4 through various microbial PAMPs including peptidoglycan and LPS [155]. NLRs like NOD1 and 2, along with NLR inflammasomes, work to detect bacterial pathogens intracellularly [155,156]. These inhaled respiratory pathogens activate these receptors, inducing the production of TSLP, IL-33, IL-25 and HMGB1 [156,157,158,159,160,161]. (**C**) Environmental pollutants like DEPs activate PAR2, resulting in the production of MMP-1 through increased intracellular Ca^2+^ concentrations via TRPV4 activation [162]. DEPs can intracellularly activate aryl hydrocarbon receptor (Ahr) and also penetrate intracellularly, causing the disruption of epithelial junctional proteins [19,163]. PM2.5 is another type of environmental pollutant that is detected by PRRs like TLR2 and 4 through microbial patterns [164,165]. TRP channels like TRPA1 and TRPV1 are also implicated in working cooperatively with PRRs like TLR4 to mediate PM2.5-induced pathology [166]. Overall, environmental pollutants induce the production of TSLP, IL-33, IL-25, IL-1α and HMGB1 through the activation of these receptors and channels [19,167,168,169]. This release of alarmins, specifically TSLP, is crucial for the development and regulation of ILC2 and Th2 proliferation, known as the ILC2-DC-Th2 axis [31]. Briefly, these alarmins can individually or synergistically activate ILC2, resulting in the production of T2 cytokines IL-4, IL-5 and IL-13 [32]. TSLP mediates the activation of dendritic cells and upregulates the expression of OX40L, which subsequently are required for the maturation of naïve T-cells into Th2 cells [19]. The downstream production of IL-5 promotes the recruitment of and production of eosinophils [170]. IL-4, on the other hand, promotes the isotype switch and secretion of IgE by B cells. This enhances the IgE-mediated immune response by upregulating high-affinity FcεRI on mast cells and basophils [94]. The degranulation of activated mast cells and basophils results in the release other inflammatory mediators, like histamine, causing bronchoconstriction, and pro-inflammatory cytokines that further drive airway inflammation. This figure was created with BioRender.com (accessed on 7 October 2024).

Inhaled allergens represent the most common and potent triggers of the inflammatory cascade in asthma. Common allergens include HDM, animal dander, pollen from grass, trees and weeds, fungal or mold spores, and cockroaches. Many allergens contain environmental lipopolysaccharide (LPS) or their own microbiota, making TLRs crucial PRRs for their detection [137]. Specifically, TLR4 and TLR2 are extensively involved in recognizing lipid segments in many inhaled allergens and driving the asthmatic inflammatory response. For example, pollen such as timothy grass pollen and components of cockroach allergens, like the crystal structure Bla g 1, possess lipid-binding proteins that trigger and activate TLR4 and TLR2, thereby promoting Th2 immunity [137,138,139]. Studies using both in vitro and in vivo models of allergic disease demonstrate that the pollen-induced activation of TLR4 leads to the production of TSLP [140] and IL-33 [141]. Similarly, the activation of TLR4 by cockroach allergens results in the release of TSLP, IL-25, and IL-33, further contributing to the inflammatory environment characteristic of asthma [142] (Figure 1A).

Up to 80% of asthmatics have allergic sensitization to HDM, such as *Dermatophagoides pteronyssinus* [171]. The TLR4 detection of some HDM components, like Der p 2, is facilitated by these allergens acting in part as a myeloid differentiation protein-2 (MD-2), an important co-signaling molecule that is required for optimal TLR4 signaling [137]. Interestingly, in vivo murine models suggest both TLR4 signaling and protease activity for the degradation of epithelial junctional proteins is crucial for triggering responses to HDM allergens [143]. This can be facilitated through the synergistic effects of MD-2 homolog HDM allergens working with HDM allergens that are proteolytic enzymes, like Der p 1 and 9 [143]. These findings provide clinical relevance to mechanisms that drive asthma pathology such as the synergistic effects of TLR4 signaling and the degradation of epithelial junctional proteins that can lead to increased epithelial barrier disruption, facilitating allergen penetration and triggering downstream inflammatory responses. While these proteases can directly impair barrier integrity by cleaving molecules that make up the epithelial tight junctions, like occludin and claudin, they can also function by triggering epithelial PARs, like PAR 1 and 2 [137,144]. PAR2, in particular, is highlighted as a key mediator in the detection of protease allergens including HDM and *Alternaria alternata*, a common fungal pathogen [148]. PAR2 is also suggested to be activated by cockroach allergens, leading to the disruption of epithelial integrity and increased penetration of allergens [142]. The detection of protease allergens, including trypsin and some fungal allergens, via PAR2 is associated with the induction of alarmins including TSLP, IL-25 and IL-33 [150,151,152]. Other PRRs including epithelial CLR dectin-1 can recognize carbohydrate motifs like β-glucans found in the inhaled fungus *Aspergillus fumigatus* as well as those found in other allergens including animal dander, pollen, cockroach allergen and HDM [137,142,145,146]. Moreover, epithelial NLRs like NOD1 are involved in recognizing Gram-negative bacteria in HDM extracts [140].

Emerging work suggests the role of various other receptors that may be involved in allergen detection and facilitating the alarmin-mediated T2 inflammatory skew. Allergen exposure poses as a stressful condition to airway epithelial cells, and thus, extracellular ATP is released as a DAMP, acting as one of many inflammatory mediators [147]. This increased extracellular ATP is associated with increased intracellular Ca^2+^ concentrations through its interaction with P2 purinergic receptors, like P2X7 and P2Y2 [147]. In vitro work on normal human bronchial epithelial cells exposed to allergens, like Alternaria alternata and HDM, have demonstrated that the ATP-mediated activation of P2 purinergic receptors induce a Th2-type inflammatory response via the secretion of IL-33, TSLP and IL-25 [147,148,153]. In addition to P2 purinergic receptors, another class of proteins that can induce the influx of Ca^2+^ in response to a variety of different stimuli is the transient receptor potential (TRP) Ca^2+^ channel [148]. While these channels function mainly in response to changes in the environment including temperature, osmolarity and oxidative processes, recent work has suggested that these channels play a significant role in respiratory diseases like asthma [172]. Airway epithelial cells express various TRP subfamilies including TRP ankyrin (TRPA), like TRPA1, and TRP vanilloid (TRPV), such as TRPV1 and TRPV4. In an in vitro and in vivo combined study using human nasal and bronchial epithelial cells and a murine model, respectively, it was shown that along with PAR2, TRPV1 and TRPV4 are intricately involved in the response to protease allergens like Alternaria alternata and HDM [148]. Specifically, protease allergens induced the release of IL-33 and ATP from airway epithelial cells via TRPV1 and TRPV4. Interestingly, this effect was attenuated by the inhibition of only TRPV1 but not TRPV4 [148]. The role of TRPV1 channels was translated over into a murine model, where its genetic deletion not only attenuated IL-33 and IL-1α production but also suppressed subsequent T2 responses [148]. In a more recent study by Ouyang and colleagues, it was demonstrated that primary bronchial epithelial cells from asthmatic donors cultured as air–liquid interfaces exposed to HDM serine proteases induced Ca^2+^ influx via TRPA1, TRPV1 and TRPV4 channels and the production of IL-33 and TSLP in these cells [149], providing clinical evidence that the activation of these epithelial channels can directly induce alarmins in response to allergens in allergic asthmatics. Overall, these studies emphasize the pivotal role of TLRs, PARs, purinergic receptors, and TRP channels in driving the production of key alarmins, suggesting that targeting these pathways could be a promising therapeutic strategy to inhibit downstream T2 responses to allergens in asthmatics. 

### 5.2. Respiratory Infections

Viral and bacterial respiratory infections are also considered to be significant triggers of asthma that play a critical role in the release of alarmin cytokines from airway epithelial cells. Respiratory syncytial virus (RSV) and rhinovirus are among the most common viral triggers of asthma exacerbations [19]. Airway epithelial cell TLRs are key receptors in the detection and subsequent response to inhaled viral pathogens. As mentioned earlier, the intracellular TLRs within endosomal compartments are the primary receptors that detect viral nucleic acids [11]. In most viral infection cases, TLR3 and TLR7 are critical TLRs in detecting viral double-strand (ds) RNA and single-strand (ss) RNA, respectively. Further viral RNA detection can also be carried out by RLRs like RIG-1 and MDA-5 [154]. The detection of viral RNA is associated with the downstream production of a variety of mediators that are involved in the initiation of the innate antiviral response [154]. In particular, it has been demonstrated that virally challenging an HDM allergic asthma murine model induces an increased expression of TSLP, IL-33, and IL-25, along with Th2 cytokines and RIG, MDA5, and TLR3 PRRs [157]. Interestingly, another study established that IL-33 in particular played a critical role in driving viral-induced asthma exacerbations, an effect that was attenuated by inhibiting IL-33’s receptor, ST2 [158]. The rapid release of these cytokines activates downstream cells like ILC2 cells, leading to the production of T2 inflammatory cytokines like IL-5 and IL-13 [173]. Various in vivo and in vitro studies have demonstrated an RSV-induced increase in HMGB1 expression [159]. Interestingly, a murine RSV model demonstrated that neutralizing HMGB1 significantly reduced BALF T2 inflammatory cytokines, suggesting that along with other alarmin cytokines, virus-induced HMGB1 production can further propagate asthmatic T2 inflammation [160] (Figure 1B). These findings suggest that similar pathways, such as the observed role of IL-33, may also be active in asthmatic individuals, providing potential therapeutic targets that could help mitigate viral-induced exacerbations.

Similar to viral pathogens, bacterial pathogens, like *Staphylococcus aureus* and *Mycoplasma pneumoniae*, can also trigger asthma exacerbations through similar mechanisms [174]. Airway epithelial cell PRRs can detect a wide variety of different bacterial PAMPs including LPS, peptidoglycan, flagellin and nonmethylated CpG DNA [155]. TLR4 can detect bacterial LPS, creating a TLR4-MD2-CD14 LPS receptor complex, whereas Gram-positive bacteria are primarily detected by TLR2 through the recognition of lipoteichoic acid and peptidoglycan [155]. The intracellular detection of bacterial pathogens is carried out by NRLs, like NOD1/2 and NLR inflammasomes, if there is a presence of peptidoglycan or bacterial DNA in the host–cell cytoplasm [155,156]. Bacterial infection, such as non-encapsulated *Haemophilus influenzae*, is associated with the transcriptional upregulation of alarmin cytokines like TSLP, IL-33 and IL-25 [156]. In vitro work on human nasal polyp mucosal tissue and epithelial cell lines exposed to *S. aureus* demonstrated a TLR2-dependent increase in the expression and release of IL-33 and TSLP, along with IL-5 and IL-13 [161]. Interestingly, another study using both in vitro human epithelial cells and an in vivo murine model demonstrated that ST2 overexpression increased the bacterial load of *M. pneumoniae* and induced pro-inflammatory responses, an effect that was broadly suppressed upon ST2 inhibition [175]. These findings provide clinical insight into how the bacterial-induced activation of the IL-33/ST2 pathway could contribute to asthma exacerbations, indicating that targeting this pathway may help in managing bacterial-triggered inflammation in asthmatic patients. Together, the activation of TLRs, RLRs, and the IL-33/ST2 pathway by respiratory pathogens service as a critical mechanism driving alarmin production and the T2 inflammatory response in asthma, emphasizing the need for further research into therapeutic strategies aimed at modulating these pathways.

### 5.3. Environmental Pollutants

The air that is breathed into the respiratory tract is often contaminated with a wide range of irritants, like diesel exhaust particles (DEPs) and particulate matter (PM), that can contribute to asthma exacerbations. Diesel combustion from vehicles constitutes a major source of pollution, where along with the release of gaseous pollutants, like carbon and nitrogen oxides, it also generates DEPs [176]. These particles are mainly composed of a carbonaceous core filled with highly toxic compounds like organic compounds, such as polycyclic aromatic hydrocarbons (PAHs), as well as metals and other pollutants [176]. Once inhaled, DEPs have the ability to dysregulate the airway epithelium by not only penetrating through and disrupting epithelial junctional proteins but also by morphologically reducing ciliated cells and inducing goblet cell hyperplasia, effectively disrupting the mucociliary clearance mechanism [163]. DEPs have been shown to induce the secretion of matrix metalloproteinase-1 (MMP-1) from human bronchial epithelial cells [162]. This secretion of MMP-1 is attributed to the organic extracts (OEs) found in DEP activating PAR2, which then further induces the influx of Ca^2+^ through the activation of TRPV4 [162]. DEP-mediated toxicity and inflammation is mainly caused by the ability of DEPs to induce oxidative stress, which is believed to be in part due to cells internalizing these particles and producing reactive oxygen species (ROS) [177,178]. Inside epithelial cells, DEPs activate the cytoplasmic aryl hydrocarbon receptor (Ahr), which subsequently induces the increased mRNA expression of TSLP, IL-33 and IL-25 [19]. Through these processes, DEPs can interact with and disrupt the airway epithelium, while also inducing the production of upstream alarmins that further activate T2 responses [1] (Figure 1C).

PM2.5 is another component of environmental pollution that plays a large role in asthma exacerbations. These fine particles, with a diameter of 2.5 µm or less, are particularly concerning as they have the ability to penetrate deep into the lungs and enter the bloodstream due to their small size, allowing them to trigger inflammatory responses. They are typically formed from fuel combustion and are composed of a complex mixture of substances including organic chemicals, metals, diesel, and coal [19]. It is suggested that epithelial cells can recognize PM through microbial patterns, like LPS through PRRs, like TLR2 and TLR4 [164,165]. In an in vitro study using primary normal human airway epithelial cells exposed to PM2.5, it was demonstrated that blocking TLR2 attenuated inflammatory response via the inhibition of IL-8 production [164]. In another study using the human bronchial epithelial cell line 16HBE, it was shown that LPS from PM also activated the TLR4 pathway [165]. Moreover, an in vivo murine model demonstrated that TRPA1 and TRPV1 played critical roles in PM2.5-induced lung inflammation and AHR, an effect that was effectively attenuated upon treatments with their respective antagonists [166]. Interestingly, pretreatments with TRPA1 and TRPV1 antagonists inhibited the activation of the TLR4/NF-kB pathway, suggesting that these channels may work collaboratively with PRRs [166], providing important clinical insights in the synergistic role of TRP channels and TLR pathways in PM2.5-induced inflammation. The interaction of PM2.5 induces the production of alarmins, as demonstrated in a study using human bronchial epithelial cells exposed to both PM2.5 and HDM, where the increased mRNA and protein expression of TSLP, IL-33 and IL-25 was observed compared to cells exposed to the either stimulus alone [167]. Moreover, there is evidence that PM2.5 can induce the production of other alarmins by airway epithelial cells like HMGB1 and IL-1α [168,169]. In vivo murine models demonstrate that asthmatic mice exposed to PM2.5 also exhibit increased levels of Th2-related cytokines in their BALF, along with lung inflammation, and hyperplasia of goblet cells, highlighting the cascading effect of these particle triggers in inducing the inflammatory phenotype in asthmatics, further supporting the link between PM2.5 exposure and the exacerbation of asthma symptoms. Together, the activation of epithelial receptors, specifically TRP channels and TLRs, by environmental pollutants constitutes a critical pathway driving alarmin production and the skewed T2 inflammatory phenotype in asthma, underscoring the need for further research into therapeutic strategies aimed at modulating these key pathways.

## 6. Effect of Alarmin Blockade in Asthma—Review of Clinical Trials to Date

### 6.1. Anti-IL-25 Biologics

Recent trials targeting key alarmins have demonstrated potential in reducing asthma exacerbations and improving lung function, highlighting the potential of alarmins as therapeutic targets. The first biologic targeting alarmin cytokines was brodalumab (AMG 827), a monoclonal antibody that blocks IL-17RA, one of the IL-25 receptors [179]. A phase 2b clinical trial for this biologic was conducted to evaluate its efficacy and safety in participants with moderate-to-severe asthma, whose asthma symptoms were not adequately controlled despite the regular use of ICSs [179]. The key findings from this trial indicated that while brodalumab was generally well tolerated with no unexpected safety concerns, it did not significantly control asthma in the treated group compared to the placebo group [179], suggesting that blocking this pathway with brodalumab may not be sufficient as a standalone treatment for improving clinical outcomes in moderate-to-severe asthmatics (Table 1). Aside from this biologic, XKH001 is a humanized monoclonal IgG1 IL-25 neutralizing antibody that has entered human trials (NCT05128409) (Table 2).

### 6.2. Anti-TSLP Biologics

TSLP plays a key role in the initiation and persistence of asthma, making it a significant therapeutic target. Tezepelumab is an immunoglobulin (IgG2λ) that complexes TSLP at the binding site of its receptor (TSLPR), inhibiting the interaction between TSLP and TSLPR. In a phase 1 proof-of-concept study, tezepelumab reduced allergen-induced bronchoconstriction, airway hyperresponsiveness and eosinophilia post challenge in participants with mild allergic asthma after three monthly intravenous injections. Additionally, tezepelumab rapidly decreased markers of T2 inflammation including fractional exhaled nitric oxide (FeNO) levels, as well as blood and sputum eosinophil counts to within normal levels [180]. This study strongly supported the further development of tezepelumab for the treatment of asthma (Table 1).

Additional clinical trials of tezepelumab administered subcutaneously included a phase 2 b study (PATHWAY) conducted at 108 sites across 12 countries, in patients with uncontrolled asthma who required high doses of ICSs and had pre-bronchodilator FEV1 measurements ranging from 40% to 80% of predicted values [181]. Tezepelumab reduced the annualized asthma exacerbation rate (AAER) compared to the placebo, irrespective of baseline blood eosinophil levels. Post hoc analyses revealed that tezepelumab decreased asthma exacerbations across all four seasons [219] and significantly improved the Asthma Control Questionnaire-6 (ACQ-6) score. Tezepelumab also reduced serum IgE concentrations, blood eosinophil counts, and FeNO levels, as well as circulating levels of periostin, a biomarker of type 2 inflammation, IL-5, IL-13, and TARC. The phase 3 (NAVIGATOR) study also demonstrated significantly reduced AAER, irrespective of the baseline blood eosinophil count, and improved scores on the ACQ-6 and the Asthma Quality of Life Questionnaire (AQLQ). The study also found a significantly improved lung function measured by pre-bronchodilator FEV1, decreased serum IgE, FeNO, and blood eosinophil levels [184]. Tezepelumab was initially designated as a breakthrough therapy for patients with severe asthma who did not exhibit a high type 2 phenotype and had previously been ineligible for any of the currently approved monoclonal antibodies. These patients are typically treated with ICSs, long-acting beta2-agonists, with or without oral corticosteroids (OCSs), and additional asthma controllers. In 2018, the U.S. Food and Drug Administration (FDA) granted approval for tezepelumab, offering new therapeutic options for steroid-insensitive asthmatic patients. The phase 3 SOURCE study recruited patients with severe asthma dependent oral corticosteroids (OCSs) receiving medium to high doses of ICSs and long-acting beta-agonists [186] to investigate whether tezepelumab could reduce the need for OCSs in these patients, given the numerous adverse effects associated with long-term oral steroid use. The results indicated no significant difference in steroid sparing between the placebo and the tezepelumab groups [220]. This negative study may be attributed to potential flaws in the study design, which was conducted during the COVID-19 pandemic. Two additional clinical trials, WAYFINDER [221] and SUNRISE [222], are being conducted with the same primary endpoint of assessing whether tezepelumab can reduce the need for OCS therapy in patients with severe asthma. Preliminary results from the WAYFINDER trial have shown a reduction in the OCS maintenance dose from 10.9 mg/day to ≤5 mg/day in 90.6% of patients who reached week 20 of the study [223]. The long-term safety of tezepelumab treatment is being assessed in the DESTINATION phase 3 clinical trial, recruiting participants who were previously enrolled in either the SOURCE or NAVIGATOR trials [187]. This study aims to investigate the safety of tezepelumab in patients with severe uncontrolled asthma over a two-year period, which is essential for establishing its role in the long-term management of severe asthma.

Mechanistic clinical trials have helped to understand mechanisms of TSLP in the pathogenesis of asthma. The UPSTREAM study demonstrated improved AHR to mannitol in combination with reduced tissue eosinophils and mast cells, suggesting that blocking the TSLP drug inhibits downstream mast cell activation and associated AHR. Furthermore, significant reductions in eosinophils in blood, sputum, and bronchoalveolar lavage (BAL) were observed [182]. The CASCADE study on patients with moderate-to-severe uncontrolled asthma demonstrated a significant decrease in eosinophil infiltration in the airways, while other cell populations, including neutrophils, mast cells, and T lymphocytes, as well as the thickness of the reticular basement membrane, were unaffected by tezepelumab [183]. This study also reported a significant reduction in AHR to mannitol in patients receiving the drug [224]. These studies collectively highlighted the multifaceted therapeutic potential of blocking TSLP. Other mechanistic trials are ongoing to investigate the effect of tezepelumab on airway hyperresponsiveness and mast cell sub-populations and the activation state in patients with mild allergic asthma [188] and to evaluate the effect of tezepelumab on nasal symptoms following allergen exposure in asthma patients with allergic rhinitis [189].

The development of ecleralimab (CSJ117) highlights the ongoing innovation in targeting TSLP to manage asthma. Ecleralimab is an inhaled anti-TSLP antibody fragment that binds to TSLP in the airways, thereby preventing the activation of the TSLP receptor and inhibiting subsequent inflammatory signaling cascades. This antibody fragment was being developed for patients with severe uncontrolled asthma using standard inhaled therapies. In a phase 2a proof-of-concept study using allergen bronchoprovocation as a model for asthma exacerbations, ecleralimab was well tolerated and significantly reduced allergen-induced bronchoconstriction in adult patients with mild asthma [190]. These results indicate that systemic TSLP blockade is effective for the treatment of asthma. There also has been a strong rationale to develop inhaled formulations of anti-TSLP, such as eclerimab, to deliver drugs to airways where TSLP is released by airway epithelial cells. In the allergen challenge model, ecleralimab significantly inhibited allergen-induced bronchoconstriction at a level similar to tezepelumab [190]. However, the clinical development of ecleralimab was halted as part of a larger decision by Novartis to terminate their respiratory drug program. Another inhaled anti-TSLP, AZD8630/AMG104, is in clinical development [191] (Table 2). Other anti-TSLPs in clinical development include GB-0895, an anti-TSLP neutralizing antibody [207], CM326, a monoclonal anti-TSLP antibody [225], SHR-1905, a humanized IgG1 kappa monoclonal antibody that inhibits TSLP [193], and TQC2731, a humanized IgG1 monoclonal anti-TSLP antibody [209]. While these other anti-TSLPs work by neutralizing TSLP directly, verekitug (UPB-101/ASP7266) is another anti-TSLP antibody currently in clinical development but works directly by targeting the TSLP receptor [194] (Table 2).

Lunsekimig (SAR443765) developed by Sanofi, is another biologic being tested for its efficacy in treating asthmatics. This biologic is a novel bispecific NANOBODY^®^ molecule that can target both TSLP and IL-13 [192]. Phase 1 clinical trials were conducted on healthy volunteers and participants with mild-to-moderate asthma to evaluate the safety, pharmacokinetics and pharmacodynamics of lunsekimig. Overall, this trial found that the biologic was well tolerated in healthy adults when given either multiple subcutaneous doses of 100 and 200 mg every two weeks or a single dose of up to 400 mg either intravenously or subcutaneously [192]. Moreover, these doses did not induce any significant immunogenicity and effectively demonstrated a linear pharmacokinetic profile while actively engaging with TSLP and IL-13. Currently, phase 2b (AIRCULES) is underway to evaluate its efficacy, safety and tolerability as an add-on therapy in moderate-to-severe asthmatics [226].

### 6.3. Anti-IL-33 Biologics

The development of anti-IL-33 monoclonal antibodies has also shown some promise in asthma clinical trials. These biologics work by neutralizing IL-33’s activity, thereby reducing IL-33-induced inflammation and downstream effects. Itepekimab (formerly known as REGN3500 and SAR440340) is a human IgG4P monoclonal antibody that specifically targets IL-33. In 2018, a phase 1 and 2a randomized clinical trial assessed the safety and efficacy of itepekimab both alone and in combination with dupilumab, another monoclonal antibody that neutralizes both IL-4 and IL-13. This study focused on patients with moderate-to-severe asthma who were undergoing treatment with ICSs plus LABA and demonstrated that itepekimab improved FEV1 and overall asthma control compared to a placebo. However, similar improvements were observed with dupilumab alone, indicating that adding itepekimab may not provide additional benefits over dupilumab. The combination therapy of itepekimab and dupilumab yielded similar improvements in %FEV1 and asthma control compared to those achieved with dupilumab alone [195,196], possibly due to a significant overlap in pathways targeted by each drug. While the results for itepekimab thus far suggest it is a safe and effective treatment option for moderate-to-severe asthma, its comparative efficacy for existing treatments like dupilumab poses questions about its distinct clinical value. A phase 1 study was initiated to evaluate the effects of itepekimab, dupilumab, and a combination of the two compared with a placebo in patients with mild asthma on sputum biomarkers, although, no results have been published from this study yet [197]. The use of etokimab (ANB020) has already demonstrated efficacy in treating adult moderate-to-severe atopic dermatitis and severe adult peanut allergy. This biologic targets IL-33, thereby potentially mitigating the inflammatory cascade associated with asthma and other allergic conditions. A phase 2a proof-of-concept clinical trial was conducted to evaluate the efficacy of etokimab in adults with severe eosinophilic asthma. The trial results showed a significant improvement in FEV1 and reductions in the Asthma Control Questionnaire (ACQ-5) score, sustained through Day 64 of the study treatment [198]. Tozorakimab (MEDI3506), developed by AstraZeneca, is an anti-IL-33 antibody currently being investigated for its efficacy in treating asthma. A phase 2a study, FRONTIER-3, has been conducted to evaluate the effects of tozorakimab in adult participants with uncontrolled moderate-to-severe asthma. This study was designed to assess the safety, tolerability, and efficacy of tozorakimab, with a particular focus on its ability to improve asthma control and reduce exacerbations in patients who have not achieved adequate management with existing treatments. The study was recently completed, and its results are anticipated to provide valuable insights into the potential benefits of targeting IL-33 in this patient population [199].

### 6.4. Anti-ST2 Biologics

Blocking IL-33 signaling can also be accomplished by targeting the receptor, IL-1RL1, also referred to as ST2. There are two forms of ST2: a soluble form and one that is bound to cell surfaces. Targeting the membrane-bound form of ST2 should theoretically produce results similar to those seen with IL-33 inhibitors; however, IL-33 also possesses chemoattractant properties that lead to cell migration and tissue damage, which might not be fully addressed by blocking ST2 alone [227]. Melrilimab (formerly known as GSK3772847) is an anti-ST2 monoclonal antibody developed by GSK Pharma. In a phase 2a clinical trial of moderate-to-severe asthma patients, melrilimab demonstrated an 18% lower chance of the loss of asthma control compared to a placebo [200]. In a study conducted in participants with moderate-to-severe asthma with allergic fungal airway disease (AFAD), melrilimab did not improve FEV1, blood eosinophil levels, or AQLQ scores from baseline [201] and was discontinued from clinical development for asthma. Astegolimab is another anti-ST2 monoclonal antibody that was developed by Genentech. In a phase 2b randomized controlled trial, ZENYATTA, astegolimab demonstrated a significant reduction in the AAER by 43% in patients with severe asthma. Although astegolimab was discontinued for asthma, this substantial decrease in exacerbation rates highlights the potential of blocking ST2 for the control of asthma exacerbations [202]. Clinical studies with astegolimab are now underway in patients with chronic obstructive pulmonary disease.

As more promising data arise for targeting upstream alarmin cytokines, several newer investigational drugs and biologics have been developed to target these alarmin cytokines or their receptors. Table 2 summarizes these investigational therapeutics that are currently in the preclinical or early clinical stages for evaluation of asthma treatment. Among them, a new class of drugs is targeting IL-33 in a unique way. IL-33 Trap is a neutralizing fusion protein that combines the ST2 receptor with the co-receptor, IL1RAcP. The objective of this innovative design is to prevent IL-33 from binding to the ST2 receptors expressed on various immune cells, thereby inhibiting its pro-inflammatory signaling [212]. The IL-33 Trap functions by effectively “trapping” IL-33 before it can engage with its natural receptor, ST2, on the surface of target cells. Doing so aims to block the downstream inflammatory cascade that contributes to asthma symptoms and exacerbations. In experimental pre-clinical models of allergic airway inflammation, the anti-inflammatory effects of the IL-33 Trap have been demonstrated, showing promise for its potential application in asthma therapy. These models have revealed that the IL-33 Trap can significantly reduce markers of inflammation and improve AHR, suggesting its efficacy in mitigating allergic responses [212].

### 6.5. Personalized Medicine for Treatment of Asthma

The development of multiple biologic therapies targeting various pathways in asthma underscores the complexity of this disease and the challenges faced in finding universally effective treatments. Despite significant advances, the diversity in patient responses highlights the need for personalized medicine approaches in asthma care. The fact that biologics targeting different aspects of asthma pathology continue to be developed emphasizes that no single treatment has proven to be universally effective for all patients. It has been estimated that 60–80% of asthma patients exhibit different responses to asthma treatments, largely due to genetic factors [228]. While some biologics, such as tezepelumab, have received FDA approval and are actively prescribed by physicians, reports persist of patients not responding adequately to these treatments. This discrepancy raises important questions about whether specific patient characteristics or immune system variations influence their response to different biologics.

Genome-wide association studies (GWASs) have played a pivotal role in uncovering numerous single-nucleotide polymorphisms (SNPs) that impact the activity and expression levels of various proteins. These genetic variations can have significant implications for drug metabolism. For instance, if SNPs occur within the genes encoding drug-metabolizing enzymes or transporters, they can alter drug disposition, potentially compromising efficacy or increasing toxicity. In recent years, the role of SNPs in determining the efficacy of inhaled corticosteroids and other conventional asthma therapies has been well documented [229,230,231].

Studies exploring the association of alarmin SNPs to asthma risk and protein production have underscored the importance of understanding genetic influences on disease pathology [232,233,234]. The pharmacogenetic landscape of biologics in asthma treatment remains largely unexplored. A noteworthy study conducted by Slager et al. provides significant insights into this area by examining the association between SNPs in the *IL-4RA* gene and treatment response to pitrakinra, a recombinant IL-4Rα receptor antagonist that inhibits the downstream signaling of IL-4 and IL-13. Their findings indicate that individuals carrying specific genotypes of the *IL-4RA* gene derive greater therapeutic benefit from pitrakinra compared to those without these genetic markers [235]. This study marks a significant step forward in understanding the relationship between SNPs and biologic response in asthma; it represents only the beginning of this critical area of research with direct application to prescribing anti-alarmin biologics in clinical practice.

## 7. Conclusions

Epithelial-derived alarmin cytokines play a critical role in the initiation of innate immune responses and are intricately implicated in the pathogenesis of asthma. This review highlights the complex interplay of epithelial-derived alarmins, particularly TSLP, IL-33, and IL-25, in orchestrating the skewed T2 inflammatory phenotype that underlies the pathology in asthma. As upstream mediators, these alarmin cytokines trigger a cascade of immune responses that result in the activation and recruitment of Th2 cells, ILC2s, eosinophils and other immune effector cells, further perpetuating the inflammatory cycle in asthma. Various triggers, including allergens, respiratory pathogens and environmental pollutants, activate receptors on airway epithelium, including TLRs, like TLR2 and 4, and TRP channels, leading to the release of alarmin cytokines. This review also emphasizes the emerging therapeutic potential of targeting these alarmins to disrupt the inflammatory pathways central to asthma. Importantly, alarmin-targeting therapies, such as biologics like tezepelumab, have shown promising efficacy in reducing asthma exacerbations and improving lung function, representing a groundbreaking shift in asthma treatment, especially for patients with severe asthma who do not respond adequately to ICS treatment. Despite these advancements, there remain significant gaps in our understanding, particularly regarding the role of less conventional alarmins like HMGB1, IL-1α, IL-36 and TL1A in asthma pathology, as well as genetic factors that predispose patients to respond to specific biologics. Additionally, it is important to note that there remain areas that require further research, including the precise role of IL-25 in severe asthma and investigating the long-term effects of IL-33 inhibitors. Moreover, variability in patient response to current biologics highlights the need for a personalized approach to asthma treatment. Altogether, the airway epithelium plays a critical role in asthma, acting both as a barrier and a source of key inflammatory mediators. The development of therapies targeting the upstream epithelial-derived alarmins represents a promising strategy for managing more severe forms of asthma, in which traditional treatments are insufficient. As research continues, it is imperative to focus on a more individualized approach to treatment, incorporating genetic and environmental factors to optimize therapeutic outcomes for asthmatic patients.

## Figures and Tables

**Table 1:** Summary of efficacy of anti-alarmin cytokine biologics in asthma clinical trials.

Drug Name	Study Name	Clinical Trial ID	Characteristics	Study Population	Main Results/Endpoints	References
IL-25						
Brodalumab	-	NCT01199289	Phase 2b, Randomized, Double-Blind, Placebo-Controlled Study	Moderate-to-severe uncontrolled asthmatic patients taking ICS	- Generally well tolerated with no unexpected safety concerns - No treatment differences observed between treated vs. placebo group	[179]
TSLP						
Tezepelumab	-	NCT01405963	Phase 1, Randomized, Double-Blind, Placebo-Controlled, Parallel Design, Multiple-Dose Study	Mild atopic asthma	- Reduced allergen-induced bronchoconstriction - Decreased FeNO - Decreased blood and sputum eosinophil counts	[180]
	PATHWAY	NCT02054130	Phase 2 Randomized, Double-Blind, Placebo-Controlled Study	Severe, uncontrolled asthmatic patients taking ICS	- Decreased asthma exacerbations - Reduced AAER - Improved the ACQ-6 score - Reduced serum IgE concentrations - Reduced blood eosinophil counts and FeNO levels. - Reduced blood levels of periostin, IL-5, IL-13, and TARC	[181]
	UPSTREAM	NCT02698501	Phase 2 Randomized Double-Blind, Placebo-Controlled Trial	Adult asthmatic patients taking ICS with or without LABA	- Improved mannitol PD_15_, - Decreased eosinophil counts in airway tissue, BAL, sputum, and blood	[182]
	CASCADE	NCT03688074	A Phase 2, Multicentre, Randomized, Double-Blind, Placebo-Controlled, Parallel-Group Study	Uncontrolled moderate-to-severe asthma	- Improved mannitol-induced AHR - Decreased airway eosinophil counts	[183]
	NAVIGATOR	NCT03347279	A Phase 3 Multicenter, Randomized, Double-Blind, Placebo-Controlled, Parallel-Group Study	Uncontrolled moderate-to-severe asthma	- Reduced AAER - Improved ACQ-6 and the Asthma AQLQ scores - Improved lung function - Decreased serum IgE, FeNO, and blood eosinophil counts	[184]
	NOZOMI	NCT04048343	A Phase 3, 52-Week, Open-Label, Multicenter Study	Uncontrolled moderate-to-severe asthma	- No new adverse events were observed	[185]
	SOURCE	NCT03406078	A Phase 3 Multicenter, Randomized, Double-Blind, Placebo-Controlled Study	OCS-dependent severe asthma	- No significant effect on OCS use - Improved number of asthma exacerbations, lung function, blood eosinophil count, FeNO, and serum total IgE concentration at week 48	[186]
	DESTINATION	NCT03706079	A Phase 3 Multicenter, Double-Blind, Randomized, Placebo-Controlled, Parallel-Group, Safety Extension Study	Uncontrolled moderate-to-severe asthma	Ongoing	[187]
	-	NCT05740748	Phase 2 Multicenter, Randomized, Double-Blind Placebo-Controlled Parallel-Group Study	Mild allergic asthma	Ongoing	[188]
	TEZARS	NCT06189742	A Phase II Open-Label Exploratory Mechanistic Pilot Study	Patients with coexisting allergic asthma and allergic rhinitis	Ongoing	[189]
Ecleralimab (CSJ117)	-	NCT03138811	A Phase 1, Randomized, Subject- and Investigator-Blinded, Placebo-Controlled, Parallel Design, Bronchoprovocation Study	Mild atopic asthma	- Reduced allergen-induced bronchoconstriction	[190]
AZD8630/AMG104	-	NCT05110976	A Phase 1, Randomized, Blinded, Placebo-Controlled Safety Study	Healthy adults and asthmatic adults on medium-/high-dose ICS/LABA	-Significant reduction in FeNO in asthmatics evident from Day 7 until end of treatment (Day 28) - Lower levels of serum IL-5 and IL-13 in treated individuals compared to placebo	[191]
Lunsekimig	-	NCT06566764	A Phase 1, Randomized, Double-Blind, Placebo-Controlled, Safety and Proof of Mechanism Study	Mild-to-moderate asthma	- Well tolerated with mild-to-moderate treatment-emerging adverse events that were similar to placebo group - Reduced FeNO and blood biomarkers (IL-5, eotaxin-3, TARC, IgE and eosinophils) - Pre-bronchodilator FEV1 improved by week 1 in treatment group and persisted through Day 29	[192]
SHR-1905	-	NCT04905602	A Phase 1, Randomized, Double-blind, Placebo-Controlled Dose Escalation Study	Mild asthma	- Safe and well-tolerated mild-to-moderate treatment-related adverse effects - Treatment reduced eosinophil, FeNO, TARC, and eotaxin-3 levels - Significantly improved FEV1	[193]
TSLP Receptor						
Verekitug (UPB-101/ASP7266)	-	-	A Randomized, Double-Blind, Placebo-Controlled, Multiple Ascending Dose Study	Mild-to-moderate asthma	- Well tolerated and safe at all doses tested - 100 mg dose resulted in 54% and 54% mean percent change from baseline FeNO and eosinophils, respectively, at week 12	[194]
IL-33						
Itepekimab (REGN3500, SAR440340)	-	NCT02999711	A Phase 1, Randomized, Double-Blind, Placebo-controlled, Multiple Ascending Dose Study	Mild-to-moderate asthma	- Improved FEV1 and overall asthma control	[195]
	-	NCT03387852	A Phase 2, Randomized, Double-Blind, Placebo-Controlled, Parallel-Group, 12-Week Proof-of-Concept (PoC) Study to Assess the Efficacy, Safety, and Tolerability of SAR440340 and the Coadministration of SAR440340 and Dupilumab	Uncontrolled moderate-to-severe asthma	- The combination therapy of itepekimab and dupilumab yielded similar improvement results to those achieved with dupilumab alone	[196]
	-	NCT03112577	A Phase 1, Randomized, Placebo-Controlled, Parallel Panel Study to Assess the Effects of REGN3500, Dupilumab, and Combination of REGN3500 Plus Dupilumab	Mild allergic asthma	N/A	[197]
Etokimab (ANB020)	-	NCT03469934	A Phase 2, Placebo-Controlled Proof-of-Concept Study	Severe eosinophilic asthma	- Improved FEV1 - Reduced ACQ-5 score, sustained through Day 64	[198]
Tozorakimab (MEDI3506)	FRONTIER-3	NCT04570657	A Phase 2, Randomized, Double-Blind, Placebo-Controlled Study to Assess the Efficacy and Safety	Uncontrolled moderate-to-severe asthma	N/A	[199]
ST2 Receptor						
Melrilimab (GSK3772847)	-	NCT03207243	A Phase 2, Randomized, Double-Blind, Parallel-Group, Multicenter, Stratified Study Evaluating the Efficacy and Safety of Repeat Doses of GSK3772847	Moderate-to-severe asthma	- Lower chance of loss of asthma control	[200]
	-	NCT03393806	A Double-Blind (Sponsor Open) Placebo-Controlled, Stratified, Parallel-Group Study to Evaluate the Efficacy and Safety of Repeat Doses of GSK3772847	Moderate-to-severe asthma with allergic fungal airway disease	- No significant difference between the placebo and drug arms	[201]
Astegolimab	ZENYATTA	NCT02918019	A Phase IIb, Randomized, Double-Blind, Placebo-Controlled, Multicenter, Dose-Ranging Study to Assess the Efficacy and Safety of MSTT1041A	Uncontrolled severe asthma	- Significant reduction in the AAER by 43% in patients with severe asthma	[202]

**Table 2:** Anti-alarmin investigational drugs and biologics in preclinical or early clinical trial stages.

Target	Name	Mechanism of Action	Route and Dosing	Current Clinical Trial Phase	Current Clinical Trial ID	References
TSLP						
TSLP Directly	CDX-622	Humanized tetravalent bispecific antibody that depletes mast cells and neutralizes TSLP	10 mg/kg intravenous (non-human primate)	Preclinical	N/A	[203]
	HZ-1127	Humanized monoclonal anti-TSLP antibody	-	Preclinical	N/A	[204]
	LQ043H	Bivalent TSLP neutralizing single-domain antibody	Inhaled	Preclinical	N/A	[205]
	MK-8226	Monoclonal TSLP neutralizing antibody	Intravenous Dose range (0.3 mg/kg–10 mg/kg)	Phase 1b	NCT01732510 (terminated with no results)	[206]
	GB-0895	Anti-TSLP neutralizing antibody	-	Phase 1	-	[207]
	CM326	Monoclonal TSLP neutralizing antibody	Subcutaneous 220 mg/2 mL of either low- or high-dose	Phase 2	NCT05774340 (Ongoing)	[208]
	TQC2731	Human IgG1 monoclonal anti-TSLP antibody	Subcutaneous Multiple doses (70 mg, 210 mg, and 420 mg)	Phase 2	NCT05472324 (Ongoing)	[209]
TSLP Receptor	BP79	Small-molecule TSLP receptor inhibitor	Topical use targeting atopic dermatitis	Preclinical	N/A	[210]
IL-33						
IL-33 Directly	HpARI (*Heligmosomoides polygrus* Alarmin Release Inhibitor)	Product secreted by murine intestinal nematode *H. polygrus* that can interfere IL-33 pathway	-	Preclinical	N/A	[211]
	IL-33trap	Neutralizing fusion protein that combines ST2 receptor with the IL1RAcP co-receptor to trap IL-33	-	Preclinical	N/A	[212]
	5H8	Monoclonal IL-33 antibody that targets the FVLHN epitope on IL-33	-	Preclinical	N/A	[213]
ST2 Receptor	HpBARI (*H. polygyrus* Binds Alarmin Receptor and Inhibits)	Protein secreted by *H. polygyrus*, which consists of two complement control protein domains that have the ability to bind and block ST2	-	Preclinical	N/A	[214]
IL1RAcP	Anti-IL1RAcP antibody	Monoclonal antibody to human IL-1R3 (also known as IL1RAcP)	-	Preclinical	N/A	[215]
IL-25						
IL-25 Directly	LNR125	Anti-IL-25 monoclonal antibody	-	Preclinical	N/A	[216]
	22C7	Human/mouse cross-reactive antibody against IL-25	-	Preclinical	N/A	[217]
	XKH001	Humanized monoclonal IgG_1_ IL-25 neutralizing antibody	Subcutaneous multiple doses (0.5, 1.67, 3.34, 5.0, or 10.0 mg/kg)	Phase 1	NCT05128409 (Ongoing)	[218]

## Data Availability

Not applicable.
